# Applications of Nanoporous Gold in Therapy, Drug Delivery, and Diagnostics

**DOI:** 10.3390/met13010078

**Published:** 2022-12-28

**Authors:** Palak Sondhi, Dhanbir Lingden, Jay K. Bhattarai, Alexei V. Demchenko, Keith J. Stine

**Affiliations:** 1 Department of Chemistry and Biochemistry, University of Missouri–Saint Louis, Saint Louis, MO 63121, USA; 2 Mallinckrodt Pharmaceuticals Company, Saint Louis, MO 63042, USA; 3 Department of Chemistry, Saint Louis University, Saint Louis, MO 63103, USA

**Keywords:** nanotechnology, emerging technologies, parenteral delivery, targeting–molecular

## Abstract

Nanoporous gold (np-Au) has promising applications in therapeutic delivery. The promises arise from its high surface area-to-volume ratio, ease of tuning shape and size, ability to be modified by organic molecules including drugs, and biocompatibility. Furthermore, np-Au nanostructures can generate the photothermal effect. This effect can be used either for controlled release of drugs of therapeutic importance or for destroying cancer cells by heating locally. Despite the enormous potential, the research on the therapeutical use of the np-Au is still in its early stage. In this review, we discuss the current progress and future directions of np-Au for therapeutic applications.

## 1. Introduction

Nanoporous gold (np-Au) obtained by dealloying is a widely researched material and has found use in a diverse range of research areas including catalysis, energy storage, biomedical, and bioanalytical applications including biosensors [1], electrodes for use as neural probes [2], and coatings for applications in drug release [3]. The tremendous interest in the usage of np-Au arose due to its many desirable properties including high effective surface area, interconnected network of nanoscale ligaments, tunable pore volume, good electrical conductivity, and ease of surface modification [4,5]. The rapidly advancing area of np-Au research is a part of the broader effort to fabricate various nanostructured forms of gold. Over the past few decades, there has been a major focus on the controlled preparation of gold nanostructures in numerous geometrical forms [6,7].

**Figure 1.**
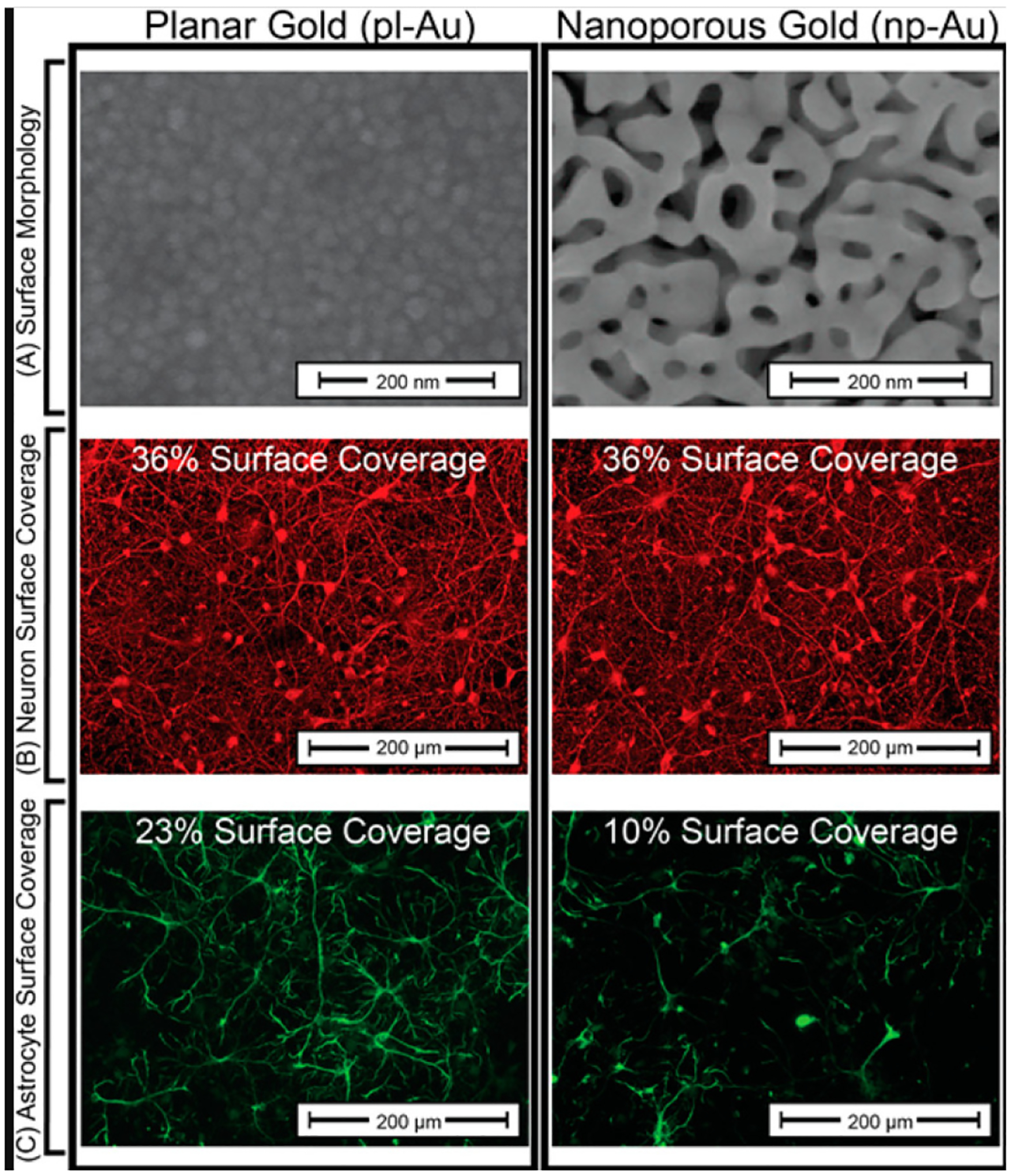
(**A**) Scanning electron micrographs of pl-Au and np-Au show marked differences in surface morphology. (**B**) Fluorescent images of β-tubulin immunopositive cells (red) show high neuron coverage on both pl-Au and np-Au. (**C**) Fluorescent images of GFAP immunopositive cells (green) indicate astrocyte coverage on np-Au that is significantly reduced compared to pl-Au. Numerical values of the cell surface coverage are provided for quantitative comparison of the fluorescent images. Reprinted with permission from ref. [2] Copyright 2017, American Chemical Society.

Nanostructured materials have shown substantial promise as multifunctional coatings from orthopedic implants to neural electrodes. The nanostructured architecture produces a large effective surface area that can improve the quality of the signal from neural electrodes by reducing the impedance at the cell-electrode interface. Nanoscale topographical features determine cell growth and differentiation for different cell types [8,9]. Astrogliosis was reduced when using nanoporous gold as a coating on neural electrodes. Np-Au suppressed the formation of scar tissue and was found to selectively decrease astrocyte coverage while maintaining high neuronal coverage in an in vitro neuron-glia co-culture model. The np-Au surface topographic features are the driving force majorly responsible for a noncytotoxic decrease in the ability of astrocytes to cover the surface of np-Au. Figure 1 shows a comparison of fluorescence microscopy images from cortical neuron-glia co-cultures grown on planar gold (pl-Au) with those grown on np-Au. The surface coverage evaluation clearly showed that the neuronal surface coverage remained constant at 36%; however, astrocyte coverage on the np-Au surface dramatically d to 10%. The dimensions of features on the np-Au surface impacts the focal adhesion of astrocytes. The inability of astrocytes to form large focal adhesion complexes on the nanostructured surface has been proposed to contribute to the decreased astrocyte coverage on these surfaces [10].

The surface nanotopography of np-Au is important for its use as an effective substrate for use in biosensors, as an interface with cells, and as a material for drug delivery. One such area of use of np-Au has been as a cell culture platform for microglia cells. Interactions between neurons, astrocytes, and microglial cells play a critical role in understanding the propagation of neuroinflammatory conditions in the central nervous system. The culture consisting of neurons, astrocytes, and microglia can be a useful tool to study neuroinflammatory pathways [11]. In a recent study, gold substrates with different porosities were evaluated for their ability to support microglial growth, and as a platform to study the morphological changes in microglial cells upon lipopolysaccharide stimulation. Microglial proliferation was hindered when np-Au monolith was used [12]. Morphologically different gold nanostructures support localized surface plasmon resonance [13].

Porous structures have a large surface-to-volume ratio and have been used for drug delivery and release applications in the pharmaceutical industry [14]. There has been a surge of interest in the fabrication of nanostructured materials and their use in cancer therapeutics, as vascular stents, and in neurological applications. The use of np-Au as a drug delivery platform has attracted growing attention recently due to the scope of tuning the pore/ligament sizes and morphology for controlling release kinetics [15]. Biomedical device coatings made up of np-Au have shown significant promise in delivery of therapeutics wherein the porous structure of np-Au has enhanced the loading capacity of small-molecule drugs, proteins with a sustained release kinetics in physiological conditions. The release kinetics have been recently monitored on a np-Au microfluidic platform with a solution flowing at a fixed rate, to attempt to model the situation of a drug eluting stent [16]. Monolithic np-Au rod has been explored for being used as an implant for delivering doxorubicin, an anticancer drug. The encapsulation efficiency was greater than 98% without any chemical modification on the electrode or the drug. The fabricated implant has shown a sustained release of the drug for 26 days in different pH conditions [17]. Drug loading and controlled release systems have been recognized as one of the main approaches to enhance drug delivery. For this application, np-Au has been widely studied as a drug delivery agent due to its excellent corrosion resistance and providing a large surface for the formation of stable self-assembled monolayers (SAMs). In an effort to generate an implantable and biocompatible drug delivery system, the usage of thiolated β-cyclodextrin (HS-β-CD) modified np-Au monolith for pH-sensitive delivery of doxorubicin (DOX) was reported. The drug release from the HS-β-CD modified np-Au was found to be pH dependent. In this investigation, encapsulated DOX with the HS-β-CD was evaluated for its release kinetics for more than 48 h in phosphate-buffered solution (PBS, pH 7.4), acetate buffer solution (pH 5.5), and bovine calf serum (pH 7.4). The release rate clearly increased when the pH was changed from physiological pH (7.4) to more acidic (pH 5.5). This resulted from the DOX’s release from inclusion complexes and an increase in solubility brought on by the protonation of the DOX’s amino group. The use of this kind of NPG implant should take into account the lower pH values, which are generally reported to be 6.84 but lower for particular tumor forms, in the extracellular tumor environment [18].

Modern medicine is taking advantage of the conventional np-Au by functionalizing its surface with drugs and targeting molecules and modifying its shape, size, and charge for selective targeting [19]. The wearable on-demand painless drug delivery system to improve therapeutic efficacy and to better manage chronic diseases like diabetes has been realized by the innovative nano-heater integrated transdermal patch that is filled with insulin. For electrothermal transdermal therapy, the patch’s design is based on the incorporation of nano-engineered heating elements on polyimide substrates. According to the study’s findings, post-coating with reduced graphene oxide allows for the encapsulation of drugs like insulin and allows for the quick response of an electrothermal skin patch made of a pattern of gold nanoholes on polyimide [20]. For the demands of a real patient, light-triggered on-demand pulsatile release from a reservoir containing highly enriched medicines has been demonstrated to be provided by versatile drug delivery devices using nanoporous membranes made of gold nanorods and dendrimers. In both static and fluidic systems, the rate of drug release was seen to be closely proportional to the rise in temperature and the amount of energy provided from a near-IR laser [21]. The long-term effectiveness of implantable drug-delivery devices depends on sustained release of the medicine and replenishment of the drug depot. An investigation was done to show how the ionic environment affected the in-plane movement of fluorescein ions through np-Au thin films (sputtering was used to deposit an Au_0.36_Ag_0.64_ (atomic%) alloy over an 80 nm thick Au seed layer and a 160 nm thick Cr adhesion layer. After dealloying the alloy-coated coverslips in nitric acid (70%) at 55 °C for 15 min [22], the np-Au film was created of various morphologies and thicknesses. The presence of halides facilitated molecular movement by lowering non-specific fluorescein (a small-molecule drug surrogate) adsorption onto the pore walls [23].

There is also a heightened interest in the application of gold-based nanostructures for the diagnosis and treatment of tumors. In this review, we will be discussing the emerging roles for np-Au in therapeutics, drug delivery, and diagnostics. The significance of surface modification and the role of different gold morphologies in drug delivery will also be described in this review.

## 2. Nanoporous Gold-Based Platforms as Future Drug Reservoirs

### 2.1. Factors Affecting the Therapeutic Efficacy

**Figure 2.**
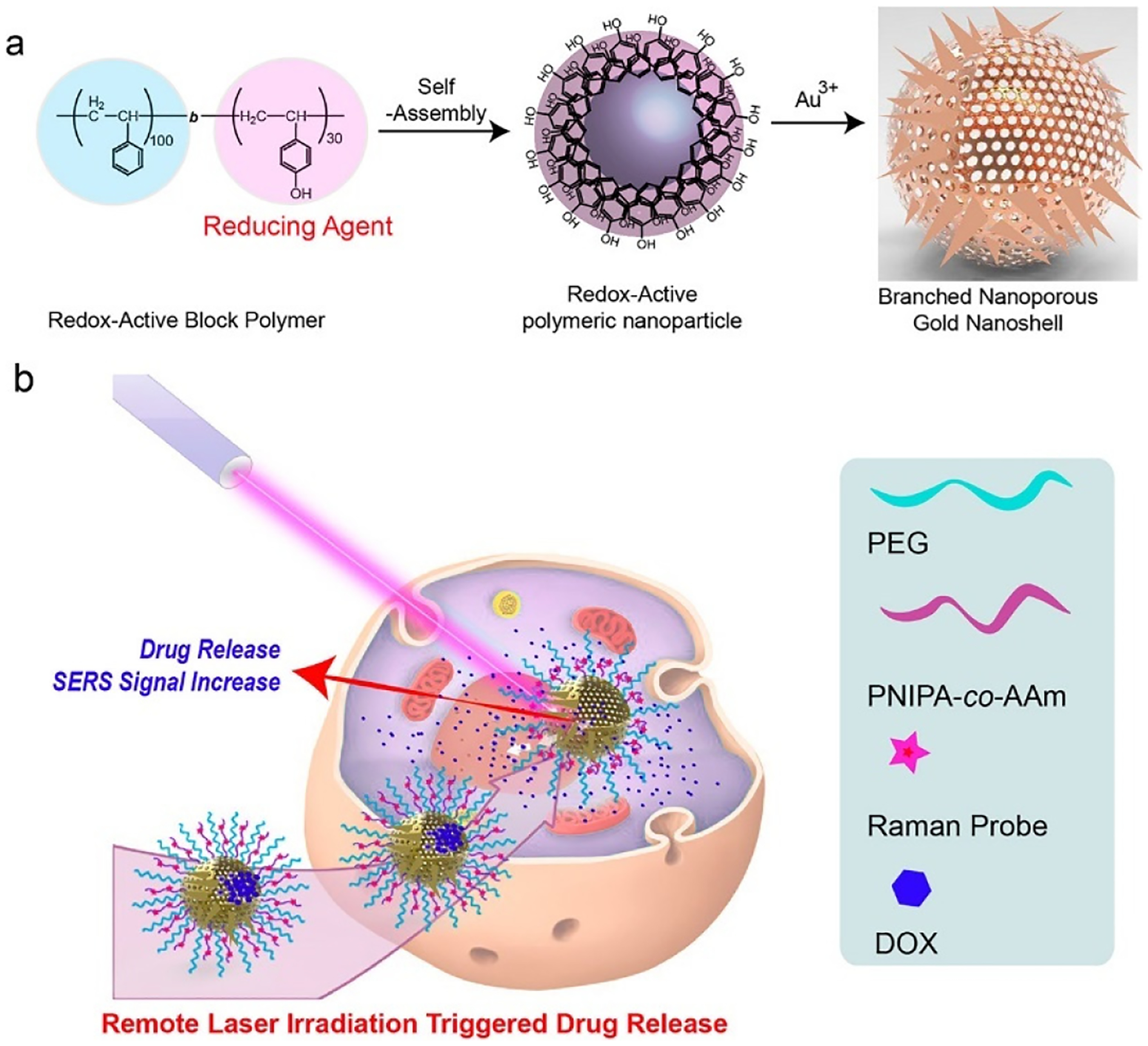
(**a**) Schematic showing (**a**) the synthesis of BAuNSP based on self-assembly of redox-active amphiphilic diblock copolymers and the localized reduction of Au^3+^ by phenol group-containing polymer NPs. (**b**) BAuNSP coated with thermoresponsive polymers for remote laser irradiation triggered drug release and optical imaging guided synergistic chemo-photothermal therapy. Reprinted with permission from ref. [25] Copyright 2017, American Chemical Society.

Nanoparticles’ size distribution, charge, and surface features are all predominantly involved in influencing their role in drug loading, release, toxicity, in vivo distribution, and stability. Pharmacokinetic and biodistribution properties are impacted by the nanoparticle size in which the particles with sizes exceeding 100 nm have shown limited clearance by the reticuloendothelial system [24]. Additionally, nanoparticle shape is an essential feature in the development of nanocarriers. The synthetic approach and the parameters involved can greatly influence the size and shape of the nanoporous structure. Different parameters including the choice of capping ligand, the solvent used, precursor concentration, reaction temperature and reaction time can all be adjusted to generate nanoporous material of ideal size and shape, meeting the biomedical demands [25]. Figure 2 shows the enhancement of the photothermal effect and photoacoustic signals of branched nanoporous gold shells (BAuNSP) coated with a thermoresponsive polymer, poly(*N*-isopropylacrylamide-*co*-acrylamide) (PNIPAm-*co*-AAm). The delivery of nanoparticles to the desired therapeutic location, in vivo stability in blood and other body fluids, attachment with target cells, and protein corona formation in vivo all depend on the nanoparticle characteristics [26]. The variable aspect ratio of gold nanorods allows the absorption wavelength to be tuned in the near-IR region and facilitates their use in imaging and potentially in the photothermal treatment of cancer. They are biocompatible, optically active absorbers and scatterers and of significant potential in the field of photodiagnostics and therapy [27]. Another interesting material comprising of gold nanoparticle-assembled capsules (GNACs) with controllable size and tunable morphology has been fabricated and applied as a hydrogen peroxide biosensor based on hemoglobin. A tandem self-assembly strategy incorporating a simple two-step mixing procedure of polyelectrolyte aggregates, formed from cationic polymers and multivalent anions into the colloidal gold solution has been used for the fabrication of GNACs with a subsequent combination of hemoglobin to form the bioconjugate, Hb-GNACs. The glassy carbon electrode modified with Hb-GNACs showed a high affinity and significant catalytic activity towards H_2_O_2_ [28]

Porous nanomaterials have gained significant momentum in their usage as agents for diagnosis and targeted therapy for cancer treatment. Further research on the influence of change in the characteristics of porous particles on their biological fate will be essential for the development of better drug delivery systems [29].

### 2.2. Mechanism of Targeting

The np-Au can be designed in zero-dimensional to three-dimensional nanostructures [30]. The delivery of the therapeutics to the desired location is based on the size of np-Au. The np-Au structures larger than a few hundred nanometers can be directly implanted near the desired location for sustained/controlled release of the drug. A submicron-thick np-Au coating was used to study the effect of halides on the release of fluorescein, employed as a surrogate for small drug molecules [15]. It was found that the interaction between halide ion and gold surface dictates the loading capacity and release kinetics. This is one of the early works marking the beginning of research on np-Au as a potential drug carrier. Recently, our lab performed in vitro experiments using np-Au milli-rod (prepared using chemical dealloying technique by leaching the reactive metals from Au alloy millirods using nitric acid) to show that doxorubicin can physically interact with the np-Au surface and has shown sustained release under physiological conditions. Additionally, we encased the DOX-loaded np-Au with rapamycin (RAPA)-trapped poly (D, L-lactide-co-glycolide) (PLGA) to fabricate an np-Au@PLGA/RAPA implant and optimized the combinatorial release of DOX and RAPA. Exploiting the effect of the protein corona around np-Au and np-Au@PLGA/RAPA has given an insight into the release kinetics of DOX which was found to be zero-order. This work proved that the np-Au-based implant has the potential to be used as a drug carrier of potential use in cancer treatment [17].

Nanostructures of np-Au smaller than 200 nm can circulate in the bloodstream for a few hours to days depending on size and surface modification. These circulating np-Au nanostructures are more likely to extravasate into the tumor region non-specifically via the leaky vessels due to the enhanced permeability and retention (EPR) effect [31]. This strategy of delivering drug molecules is more prominent for treating tumors that are not easily accessible. However, the EPR effect by itself may not be sufficient for treating most cancers due to the low accumulation of the drug. The low accumulation in the tumor could be because of clearance of nanostructures by the mononuclear phagocyte system, filtration in the kidney, or slow extravasation into normal tissues [31]. Additionally, the aberrant vasculature, increased interstitial fluid pressure, and deregulated extracellular matrix components in the tumor microenvironment work against the EPR effect. These characteristics make it difficult to deliver nanomedicines throughout the tumor in adequate numbers and uniformly, which affects sensitivity and specificity as well as treatment efficacy [32]. For the therapeutic or diagnostic substance to be delivered effectively to the tumor using the EPR effect, several things must be considered. They include blood pressure, fluid and solid stresses, nanoparticle size and shape, tumor perfusion, vascular permeability, interstitial penetration, cancer type, mononuclear phagocyte system (MPS) activity, retention of the diagnostic and therapeutic agent in tumor tissue, tumor tissue lymphatic drainage function, and others [33]. Different strategies have been employed to overcome this problem so that following extravasation, they aggressively bind to particular cells. This can be done by using a variety of conjugation chemistries to attach targeting agents, such as ligands—molecules that can bind to particular receptors on the cell surface—to the surface of the nanocarrier [34]. The use of certain ligands (antibodies or peptides) that can be grafted on the surface of a nanomedicine and precisely bind to overexpressed receptors at the target site is necessary for active drug targeting [35]. In comparison to normal organs, the EPR effect offers only modestly increased specificity and less than a 2-fold increase in nanodrug delivery. There have been reported on several interesting strategies for improving the EPR effect by getting around various obstacles to nanodrug delivery into tumors. These include actions to manage cancer-associated fibroblasts in the tumor microenvironment as well as efforts to control vasculature, regulate permeability, physically disrupt vessels, and act on the tumor microenvironment [31].

**Figure 3.**
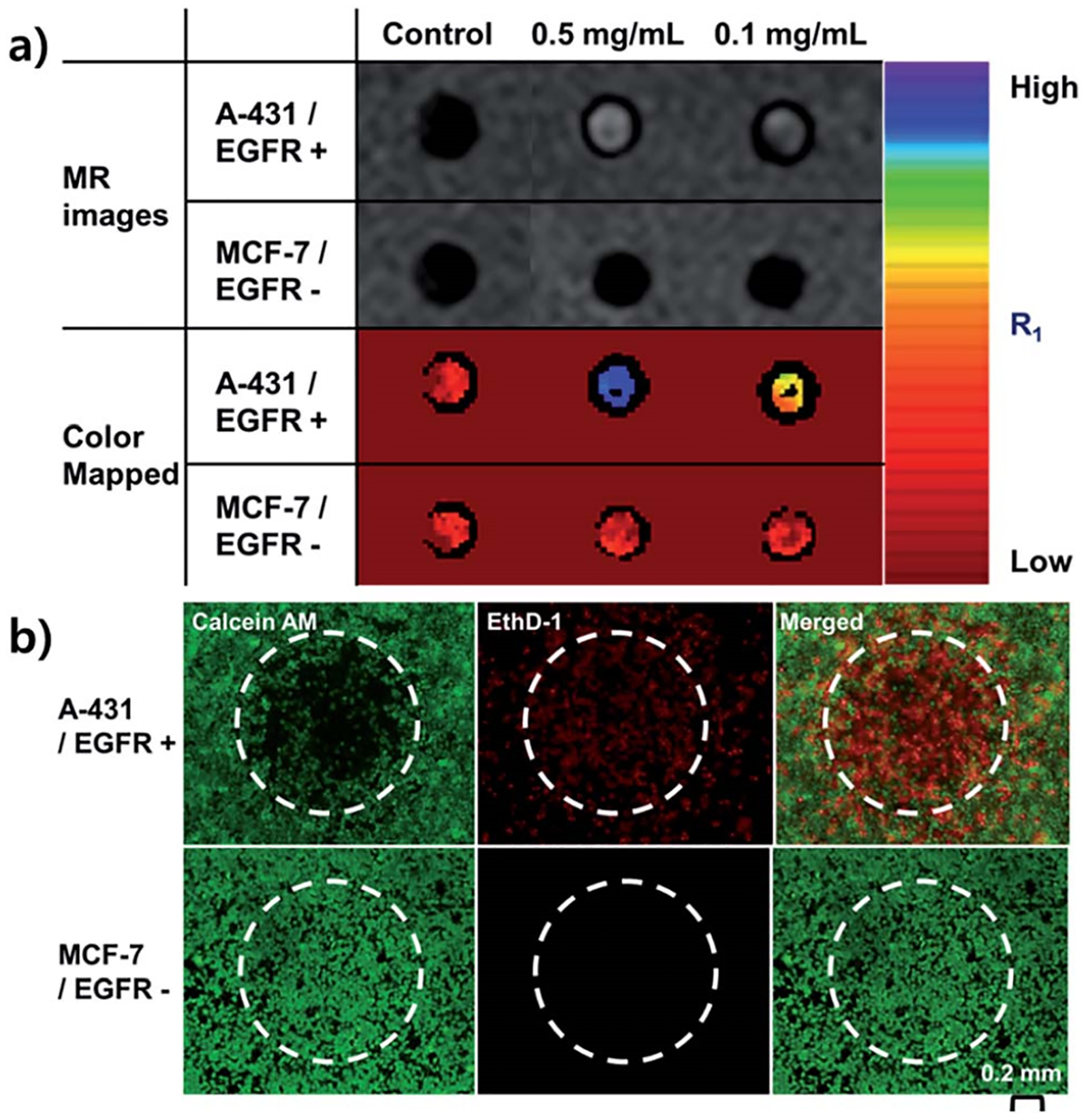
(**a**) T1-mapped and respective color-mapped MR images of A-431 (EGFR+) and MCF-7 (EGFR) cell lines after treatment with various concentrations of np-Au nanoparticles (0.5 and 0.1 mg mL^−1^). (**b**) Fluorescence microscopy images of A-431 and MCF-7 cells stained with calcein AM and ethidium homodimer-1 (EthD-1) after treatment with np-Au nanoparticles for 2 h followed by NIR laser irradiation for 10 min (808 nm, 25 W cm^−2^). White-dotted curves represent the location of the laser beam. The scale bar represents 200 μm. Reprinted with permission from ref. [36] Copyright 2011, American Chemical Society.

Another strategy includes guiding the drug load np-Au nanowire to the target location using ultrasound [14]. Once the drug-loaded nanostructures enter the local environment of the tumor the release of the payload can be controlled by NIR irradiation [14]. The temperature of the tumor environment can also be raised to heat-kill tumor cells by irradiating np-Au nanoparticles with a powerful NIR laser. It was shown in a mouse model that simply modifying the np-Au surface with mercaptosuccinic acid (MSA) can significantly increase the release of doxorubicin under the irradiation of light. The repetitive Ag-AgAu nano-segments and subsequent selective Ag phase etching was used to generate the np-Au NPs using templated pulsed electrochemical deposition. The final np-Au NPs had a typical interior porous structure with a gap size of approximately 15 nm. To modulate the chemical binding force at the interface, mercaptosuccinic acid (MSA) and mercaptopropionic acid (MPA) were added to the surface of np-Au NPs. Among the MPA modified and bare np-Au NPs, the MSA modified np-Au NPs demonstrated the superior light-triggered drug release performance for doxorubicin [36]. Lee et al. introduced the facile synthesis of mono-disperse, mesoporous gold nanoparticles (MPGNs) using acidic emulsion method. Hydrochloric acid was dissolved in an aqueous solution with aniline monomers. By injecting an aqueous HAuCl_4_ solution while the emulsion was still in motion, the metastable aniline emulsion was fixed. After the reaction was finished, a 1 M NaOH solution was used to clean the produced nanoparticles. Three rounds of centrifugation with 1-methylpyrrolidone were performed on the MPGNs (NMP). The surface of as-prepared np-Au was modified with a therapeutic antibody, cetuximab, and loaded it with gadolinium, an MRI contrast agent, for the simultaneous diagnosis by magnetic resonance (MR) imaging and treatment by photo-thermal ablation [36]. Figure 3a shows T1-mapped and respective color-mapped MR images of A-431 (EGFR+) and MCF-7 (EGFR−) cell lines after treatment with 0.5 and 0.1 mg mL^−1^ np-Au nanoparticles. When both cell lines were irradiated with NIR laser after treatment with np-Au nanoparticles for 2 h and stained with calcein AM to detect live cells (green fluorescence) and ethidium homodimer-1 (EthD-1) to detect dead cells (red fluorescence), a vivid red spot was observed at the center of A-431 cell plates likely due to cell death in response to NIR irradiation [36], Figure 3b.

### 2.3. Application to Neurological Conditions and Mental Health

In the United States, Alzheimer’s disease (AD), a neurological illness, is now the fifth-leading cause of death for the elderly. The pathologic characteristics of AD include Aβ-fibrils and neurofibrillary tangles. The development of neurofibrillary tangles, which are composed of misfolded aggregates of tau proteins connected with internal microtubules, and senile plaques, which are composed of external amyloid-(Aβ) peptides, is supported by increasing amounts of data. Preventing the buildup of amyloid beta (Aβ) peptides is one potential strategy for treating AD, and gold nanoparticles have been researched as potential anti-Aβ therapeutics [37]. Due to their excellent biocompatibility, simple functionalization, and possible capacity to traverse the blood–brain barrier, gold (Au) NPs are among the NPs that are thought to be useful nano-chaperones to inhibit and redirect Aβ fibrillization [38]. According to studies, the size, surface chemistry, and electric charge of Au NPs can all affect their capacity to prevent Aβ-aggregation. The degree of inhibition is influenced by NP surface chemistry and size, but NP ability to change aggregate morphology is defined by electric charge [39,40]. In vitro, compounds with surface chirality and helix shape can prevent Aβ aggregation in an enantioselective manner. Therefore, it is anticipated that adding chiral D-glutathione (GSH) ligands to gold NPs will provide them exceptional stability and chiral recognition of Aβ along with enantioselective suppression of Aβ fibrillation in one of the recent studies [38]. Okadaic acid (OA) produced an AD model that resulted in neuroinflammation and oxidative stress. It was interesting to note in another study that the anti-inflammatory and antioxidant qualities of AuNPs have lessened the oxidative stress (sulfhydryl and nitrite levels) brought on by OA in some brain regions. As long-term AuNP treatment reduced the neuroinflammation, control of mitochondrial function, and poor cognition generated by an AD model, AuNPs may be a viable treatment for neurodisease brought on by these variables [41]. In response to the pressing need, a label-free ultrasensitive clinical lab test for AD has been developed. This test is crucial for the current drug regimens. Using a monoclonal anti-tau antibody coated gold nanomaterial for the ultrasensitive and selective detection of tau protein AD biomarker at a 1 pg/mL level, a two-photon Rayleigh scattering (TPRS) assay has been developed. Two-photon scattering properties have been tracked using the hyperRayleigh scattering (HRS) method. It was shown that when anti-tau antibody coated gold nanoparticles (gold nanoparticles were prepared by adjusting the proportions of HAuCl_4_. 3H_2_O and sodium citrate, using sodium borohydride approach. The produced gold nanoparticles were spherical and had a diameter of 4 nm) were coupled with tau protein concentrations of 20 pg/mL, the two-photon scattering intensity increased by almost 16 times. The bioassay has proven to have a short response time from protein binding through detection and analysis [42]. The efficiency of the dot-blot immunoassay is combined with the high affinity of biotin and streptavidin in the creation of an Au NP-based dot-blot immunoassay for the detection of Aβ in complex biological materials. When compared to the well-established Aβ detection methods, this technology offers a wide dynamic range and reasonable sensitivity [43]. A dual-readout (colorimetric and fluorometric) test for acetylcholinesterase (AChE) has been discovered utilizing gold nanoparticles coated with Rhodamine B. The assay has been used to monitor AChE levels in the cerebral fluid of transgenic mice with Alzheimer’s disease due to its high sensitivity and specificity [44]. A role for np-Au nanoparticles in the future seems highly probable.

Schizophrenia is another neuropsychiatric condition that affects the central nervous system (CNS), and it is characterized by aberrant fluctuations in dopamine levels. Nanotherapeutic strategies have aided in addressing the issues with CNS medication delivery. In the past, ethyl cellulose microcapsules containing thioridazine were made using gold nanoparticles [45]. The surface modification of the Au NPs can increase their bioavailability. Iswarya et al. created a dopamine detection technique employing gold capped with L-histidine (AuNP). A change in the nanoparticles’ surface plasmon resonance (SPR) was used to track how the surface-modified Au NPs interacted with dopamine [46]. By 2030, depression is expected to afflict more than 300 million people worldwide, making it the most common debilitating ailment. Variations in the expression of glucocorticoid receptors (GRs) can be used to forecast specific cognitive abilities. GRs are also an important marker for assessing the efficacy of various treatments. A cutting-edge electrochemical biosensing technology has been created that allows for the precise and sensitive detection of depression biomarkers. By combining amino-ion graphene oxide (IL-rGO) and amino acid-coated gold nanoparticles (AA-AuNPs) using a green production method, electrochemical signals are noticeably amplified [47]. The use of gold nanoparticles as an antidepressant therapy strategy has been studied. Because they require less frequent administration and are more effective, nano-based formulations are becoming more popular [48].

## 3. Emerging Biomedical Applications of Nanoporous Gold-Based Structures

Nanoporous gold (np-Au) is a metallic structure with pores and ligaments on the nanoscale. Since these particles can be considered as a combination of nanomaterials, inert metals, and nanoporous framework, np-Au provides a wide range of applicability to the field of biomedicine [49]. Due to quantum mechanical principles, nanoparticles with diameters between 1 and 10 nm (between the size of molecules and that of bulk metal properties) exhibit electronic band structure. Nanoparticles can engage in quantum tunneling in this small size range. The physical characteristics that result rely significantly on the particle size, interparticle spacing, kind of organic shell that surrounds them, and shape of the nanoparticles. They are neither those of bulk metal nor those of molecular compounds. A few “last metallic electrons” are employed in nearby particle tunneling [50]. Tunneling effects start to interfere with the interaction between the surface plasmons when the interparticle distance is less than 1 nm, according to theoretical research by Nordlander and colleagues. The interaction between surface plasmons is disrupted by a quantum tunneling phenomenon when the interparticle distance (d) is smaller than 1 nm, which results in the red shift absorption. Tunneling of electrons to and from immobilized biomolecules may enhance the efficiency of biosensors if the biomolecule interacts with gold nanoparticles or structural features in this size range.

There is additional research being done using gold nanoparticles as biological probes. It can be mixed with a variety of biological macromolecules, including nucleic acids, heavy metal ions, and protein, thanks to its unique optical features, macroscopic quantum tunneling effect, surface effect, and strong biocompatibility [51,52]. Nanoscale pores and framework provides enhanced physical, chemical, and biological activities due to their nano-size, enhanced surface, and quantum tunneling effects. The increased surface area also works for the betterment of the adsorption capacity of np-Au by providing more binding surfaces of biomolecules. The porous structures also play a vital role in the transfer of biomolecules through their increased permeability which can catalytically help to increase the reaction rates too [53]. Better electrical conductivity and the energy absorption capacity of np-Au is another main characteristic that facilitates the transfer of electrons which makes it more important for biomedical applications. In addition, the tunability of np-Au in case of size, shape, and pore, makes np-Au more practical in this applied field [54]. Due to these special characteristics, applications of np-Au have been increasing in recent years in the field of biomedicine, such as biosensing, drug delivery, and catalysis. Biosensing has become an important part of research for the analysis and detection of biomedical elements due to its necessity-driven demand. The challenges in the biomedical field due to the surge of known and unknown biological elements are growing continuously thereby increasing the demand for diverse types of biosensors [55]. Researchers in recent years are focusing on the development of simple, low-cost, real-time, and efficient biosensors. For these purposes, np-Au has proven its standing as a promising tool with its unique and excellent characteristics. Different studies have been done to fabricate np-Au-based biosensors using its various forms such as bare np-Au, surface-functionalized np-Au, shape-controlled np-Au, and other np-Au with hybrid structures [56].

### 3.1. Role of Pore Size and Ligament Width

**Figure 4.**
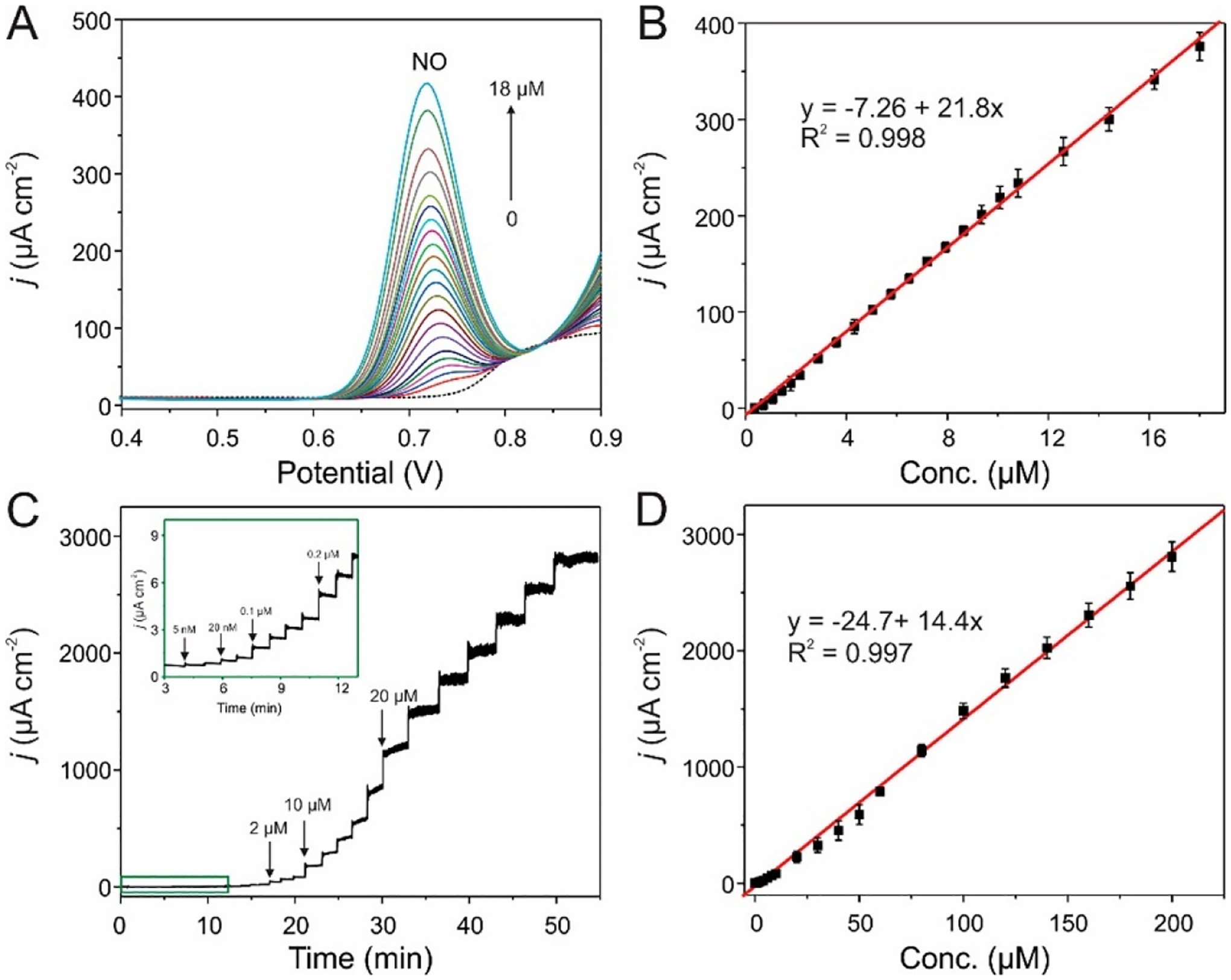
(**A**) DPV responses and (**C**) amperometric responses of the HNG microelectrode for the analysis of different concentrations of NO in 0.1 M of PBS (pH 7.2). The inset in panel (**C**) is the enlarged amperometric responses of NO in the low-concentration range. The applied potential in panel (**C**) was 0.80 V. (**B**,**D**) The corresponding calibration plots. Each point was expressed as mean ± standard deviation (*n* = 3). Reprinted with permission from ref. [64] Copyright 2017, American Chemical Society.

Cancer, diabetes, and epilepsy are just some of the diseases treated and managed presently using powerful technologies involving implantable drug delivery platforms. Drug delivery implants need to show a sustained release rate that continues under biofouling conditions. These implants may also need to respond as desired to internal or external stimuli [57]. Some of the attractive properties of np-Au include biofouling resilience, compatibility with microfabrication processes, bicontinuous open-pore structure, and facile surface functionalization [23,58]. During the past few years, these attributes led to growth in the use of np-Au in biomedical applications including drug delivery, short nucleic acid detection, neural electrodes, and drug-eluting coatings. Mechanistic details have shown the key role of surface area and pore morphology in controlling the loading capacity, and release kinetics of small molecules desorbed from the pore walls and their way out through the porous network of np-Au [3,15]. There is a great potential to tune the pore size in the dealloyed np-Au membrane. Recent work has exploited np-Au semipermeable membranes with pore size of 800 nm in cell encapsulation therapy (CET) for the treatment of endocrine and metabolic diseases. The diffusional efficiency of the np-Au membrane was investigated, and it was found that adequate diffusion of substances for cellular metabolism was sustained by the np-Au membrane [59]. The detection of the biological entities using bare np-Au is mainly based on the catalytic activity of np-Au to facilitate the electron transfer between np-Au and the target biological molecules. The resulting redox peaks obtained from the catalytic interactions were used as a tool for biomolecule detection. Np-Au prepared by simple alloying and dealloying method by Z. Liu et al. (2009) was directly used to detect p-nitrophenol (p-NP) by monitoring its redox behavior using cyclic voltammetry (CV) [60]. A good sensitivity and selectivity of the detection method was reported with a linear detection range of 0.25 to 10 mg L^−1^. A research group of H. J. Qiu et al. (2009) used np-Au-modified simple glassy carbon electrode (GCE) to detect dopamine using differential pulse voltammetry (DPV) with a limit of detection of 17 nM [61]. Glucose detection was also studied using a simple np-Au electrode in an amperometric technique by Y. Xia et al. (2011) [62]. They were able to quantify the glucose present in a linear range of 10 μM to 11 mM with a sensitivity of 66.0 μA mM^−1^ cm^−2^ and a detection limit of 8.7 μM. A highly sensitive np-Au biosensor was also used to detect the glucose uptake of skeletal muscle tissues fabricated by R. Obregon et al. (2013) which was able to respond in a wide concentration range of 1 to 50 mM, with a limit of detection of 3 μM [63]. An electrochemically prepared three-dimensional hierarchical np-Au was used for electrochemical sensing of nitric oxide (NO) by Z. Liu et al. (2017) [64], which was able to detect NO with high stability. The reported limits of detection were 18.1 ± 1.22 and 1.38 ± 0.139 nM with DPV and amperometry (Figure 4), respectively.

Despite this wide range of applications, biosensing using bare np-Au is limited to very few numbers of molecules due to the specificity and vast diversity of biological compounds. Therefore, many np-Au biosensors modified with other sensitive materials and biomaterials have been developed. Rough and activated noble metals possess enhanced sensitivity in comparison with their smooth counterparts. The np-Au films have shown higher roughness, a greater number of binding sites on the surface, and better electron transport leading to an elevated response in the field of catalysis [65]. Design and construction of hierarchical nanoporosity to gold nanoparticles (Au NPs) through controlled dealloying of Au-Cu alloy nanoparticles have been tried recently. The dealloyed structure exhibited 3D open surface structures, large specific surface area, and controlled drug loading and release [66]. A hierarchical 3D network of mesoporous Au modified with cytochrome c has been employed to target superoxide anions released from skeletal muscle tissues. Light triggered drug releasing performance of surface-engineered nanoporous gold nanoparticles (np-Au NPs) has been studied recently. The np-Au NPs were used as the host carrier for doxorubicin and the drug was released under light irradiation of the host. The carrier was prepared by applying templated pulsed cyclic chronopotentiometry with subsequent removal of the template and Ag phases. The surfaces of nanoparticles were modified with mercaptopropionic acid (MPA) and mercaptosuccinic acid (MSA) to control the chemical binding force at the interface. It was found that the MSA-modified np-Au NPs worked well for the controlled release of doxorubicin upon light irradiation [67].

### 3.2. Plasmonics-Based Applications

Plasmonic metal nanostructures have numerous uses in fields including optics, medicine, and catalysis. Their composition, configuration, environment around nanostructures, shape, and size have a major impact on their plasmonic characteristics, such as surface plasmon resonance (SPR) and localized surface plasmon resonance (LSPR). Due to the distinctive 3-dimensional bicontinuous nanostructure with a significant surface area, strong catalytic activity, and tunable plasmonic resonance, nanoporous gold (NPG) has recently received a lot of attention [68]. It is believed that a key factor in LSPR sensing and surface-enhanced optical phenomena like surface-enhanced Raman scattering SERS and surface-enhanced fluorescence is the enhanced electromagnetic (EM) fields of LSPR excited in the ligaments. By changing the morphology of porous nanostructures such the pore and ligament size by dealloying time and thermal annealing, it is possible to achieve limited tunability in plasmonic resonance [69,70]. By dealloying ultra-dilute Au-Ag alloys with a low gold content of 1–5% at.%, an ultralow density nanoporous gold (ULDNPG) with better plasmonic photocatalytic SERS performances was created. To achieve the dealloying of such diluted solid solutions, a sandwich dealloying strategy was developed. Excellent SERS characteristics of these ULDNPG structures include high sensitivity, good repeatability, and low cost [71]. Small molecule label-free sensing has been accomplished using the morphological characteristics of NPG as a capturing scaffold. Recently, DNA topologically functionalized plasmonic nanostructures were used in SERS sensing systems. Target molecules, such as malachite green, can be attracted to NPG disks (NPGD) surfaces by stacking and electrostatic forces by using guanine quadruplex (G4) moieties. The collected molecules generated a remarkable SERS signal because of the high-density plasmonic hotspots on NPG disks [72,73]. A microfluidic device with NPGD monolithically embedded inside has been used to produce a microfluidic SERS sensor. The three-dimensionally distributed nanoscale pores and ligaments in the NPGD, which appear as high-density SERS hot-spots, are what contributed to the enhanced surface area. Further ensuring extensive coverage of these hotspots on the microchannel floor are high-density NPGD arrays [74,75]. For the first time, dual modality sensing (chemical and refractive index) at different molecule path lengths has been demonstrated using surface-enhanced near-infrared absorption (SENIRA) of overtone and combination bands on NPG disc substrates. Beginning with the DC sputtering of the Au:Ag (30:70) alloy over the glass coverslip, NPG disk arrays bound to substrates are created. After that, a monolayer of polystyrene (PS) beads was applied to the alloy film’s outside. Oxygen plasma etching was used to shrink and isolate the PS beads, and then Ar plasma etching was used to promote alloy disk formation on the glass surface. By dissolving in chloroform, the residual PS beads on top of the metal disks were removed. The disks underwent a 1 min dealloy in 70% nitric acid and a 2 min DI water wash [76]. A consistent and replicable SERS substrate with a strong response at low detection limits has been created. The substrate was made up of stacked, ultrathin NPG layers and was used to find molecules in liquids or gases at low concentrations. Gold and copper targets are used in the thin-film deposition process on monocrystalline (100) silicon substrates. A 120 nm thick chromium film is first deposited, followed by a 50 nm thick pure Au protective coating, all before the Cu/Au multilayer deposition. To etch the copper from the stacked multilayers for 300 min, concentrated nitric acid is utilized. Alternative copper and gold stacked nanolayers have a strong anisotropy in their morphology along the perpendicular axis to the substrate surface as a result of copper chemical etching. The gold nanoporous film’s SERS behavior is directly influenced by its morphology. This is due to the morphology’s crucial spacing between nearby gold ligaments, which is one of the key factors in the development of hotspots [77]. NPG films have demonstrated outstanding sensitivity in portable sensing devices without the need for complicated production techniques. The co-localization of optical energy and analytes in the pores, which promotes an improved light-matter coupling, is related to the sensing mechanism. As a result, the NPG film exhibits a sharp change in reflectivity and a considerable spectrum shift in the effective plasma frequency when molecules are adsorbed in the pores. The analyte can be found by observing the reflectivity in the spectral area near the plasma frequency, or more specifically, the plasma edge [78].

### 3.3. Surface Functionalized np-Au-Organic/Inorganic Materials

Responsive materials are being created by their surface functionalization with moieties which helps them to adapt to the external environment changes including temperature, pH, and fluid composition. Smart nanomaterials range from polymer-based nanoparticles to inorganic metal, metal oxide, and metalloid nanoparticles [79]. The combination of polymer and noble metal nanoparticles not only provides a large surface area and mechanical strength but also limits the accidental release of nanoparticles into the environment. This contributes to the application in medical fields: as a drug delivery system, and for tumor hyperthermia [80]. It has been seen that the combination of freestanding and nanoporous microelectrodes with molecularly imprinted polymer (MIP) functionalization offers great features. Electrochemical detection of metronidazole (MNZ) at pharmaceutical dosages and in real biological samples like fish tissues was successfully achieved by using a freestanding Au-Ag alloy microrod electropolymerized with MIP in the presence of MNZ. Further, MNZ was extracted using H_2_SO_4_ to generate the 3D open nanoporous structure which displayed both specificity and anti-interference properties [81]. Even though the sensitivity was enhanced, the selective and specific detection of biomolecule demands tremendous effort for the betterment of a biosensor. The np-Au biosensors functionalized with biological molecules such as enzymes and immune complexes provide more selectivity and specificity towards the detection. Enzymes have been a major component chose for use in the development of biosensors because of their active catalytic activity and great selectivity towards biomolecules. The ease of enzyme immobilization on the surface of various forms and sizes of np-Au makes the construction of an np-Au-based enzymatic biosensor more convenient and important. Many studies about enzyme-based np-Au biosensors have mentioned the use of several types of oxidases, reductases, and hydrolases for the detection of small molecular compounds and other important biological molecules such as H_2_O_2_, glucose, and cholesterol. The electron mediating and prosthetic groups have always been the important materials for np-Au-modified electrode modification. A. Zhu et al. have used a cytochrome c/np-Au/indium tin oxide (ITO) electrode system for electrochemical detection of H_2_O_2_ [82]. R. B. Sadeghian et al. have also reported the use of cytochrome c to functionalize np-Au electrodes for the detection of superoxide from skeletal muscle cells [83]. A. K. M. Kafi et al. have immobilized hemoglobin (Hb) onto the np-Au-modified titanium substrate to detect H_2_O_2_ [84]. C. Wu et al. have mentioned the selective oxidation of phenol and aromatic amines using horseradish peroxidase (HRP) modified np-Au/glassy carbon electrode (GCE) to analyze the real seawater sample [67]. Many pieces of literature also show the use of these enzyme functionalized np-Au-based biosensors for monitoring the human body metabolites such as cholesterol. A. Ahmadalinezhad et al. synthesized a cholesterol detecting biosensor with the immobilization of cholesterol oxidase, cholesterol esterase, and HRP on an np-Au-Ti matrix [85]. Similarly, many researchers have investigated the monitoring of blood glucose using glucose oxidase (GOD) immobilization on np-Au-modified electrodes. H. J. Qiu et al. and his research group incorporated GOD into a matrix of np-Au to analyze glucose [86]. T. J. Li et al. immobilized the GOD on a Prussian blue (PB)-modified np-Au electrode to detect glucose on blood samples [87]. Despite this much research about GOD-immobilized np-Au-based biosensors, not many commercial applications have emerged and achieving this will require further improvements in the stability and long-term storage of these biosensors. Another widely studied biosensor type using surface-functionalized np-Au is immunosensors because of their higher specificity, selectivity, sensitivity, and stability for detecting the target molecules. As compared to the enzymes, these complexes are known to be more specific towards their target. These immunosensors are a combination of general immunoassay with biosensing technology. The np-Au-modified immunosensors are based on the binding and recognition through antigen-antibody or receptor-ligand interactions. As compared to traditional immunoassays, these get the benefits from the use of np-Au to make it more conducting and biocompatible with increased specific surface area. These immunosensors have been used to diagnose cancer and to detect other viruses such as influenza A [88,89].

### 3.4. Hybrid Structures Involving np-Au

Electrochemical biosensors have been widely used in clinical research for recognizing biological analytes through a catalytic or binding event occurring at the electrode’s interface [90,91]. Tremendous demand for enhancing charge transport in the biosensors to significantly increase its sensitivity and reliability along with faster response times have stimulated intensive research on developing versatile materials with ultrahigh activities towards catalysis. For this reason, composite materials are being designed to combine highly electrocatalytic materials with a conductive material [92]. Hybrids with two-dimensional and three-dimensional nanostructures have attracted great interest due to the unique structural features arising from combining different materials. Early diagnosis and effective treatment of conditions like Alzheimer’s are very critical to reducing the impaired cognitive functions in an aging population. It has been seen that acetylcholinesterase (AChE) inhibitor drugs could act as a potential treatment option. A simple and rapid nanoporous gold film (NPGF) based biosensor platform has been developed for the detection and inhibition of enzymatic reactions of AChE. The electrode was prepared using anodic stripping of Zn from Au-Zn alloy (For this, a multi-cyclic alloying/dealloying process was carried out from 1.45 V to −0.70 V in an electrolyte of benzyl alcohol containing 1.6 M zinc chloride for 30 cycles under a scan rate of 10 mV/s) and was further surface-modified using reduced graphene oxide nanosheets (RGO) containing tin oxide dopants. The nanocomposite has been shown to facilitate mass transport at the NPGF electrode’s surface thereby increasing the electrode’s selection sensitivity. The fabricated electrode has shown high detection capability of 8 pM towards fasciculin, a natural inhibitor of AChE [93]. Recently, a hybrid electrode with ultra-thin, ultra-light, and flexible characteristics was created with graphitic carbon nitride (g-C_3_N_4_) nanosheets that have been electrochemically deposited on the surface of NPGF. The hybrid electrode has shown a striking enhancement of supercapacitive performance (specific capacitance of 440 F g^−1^ at 2A g^−1^ in 0.5 M Na_2_SO_4_ solution). The superior property has been attributed to the strong interfacial effect between the defected gold atoms of np-Au and g-C_3_N_4_ [94]. Metal oxides supported 3D hierarchical porous np-Au/Ni foam electrode has shown exceptionally high catalytic activity resulting mainly from its open and porous structure facilitating the mass transport and charge transfer [95]. In recent times, a versatile electrochemical sensor has been created by using a two-step coating technique of integrating np-Au leaf and molecularly imprinted polymer (MIP) to analyze warfarin sodium (WFS) in human blood. The hybrid electrode was designed using the Ag/Au alloy leaves which were dealloyed using 65 wt.% nitric acid and affixed to the planar gold electrode surface (4 mm in diameter). After drying under an infrared lamp, the modified electrode was kept in a solution containing functional monomer (resorcin) and WFS. The sensor fabrication was completed by immersing the electrode in 0.1 M NaOH to remove the template [96].

Scientists are working intensively in the field of cancer diagnostics and therapeutics. In most tumor cells the overexpression of telomerase has been found responsible for uncontrolled cell proliferation. Due to this reason, scientists are working toward creating sensitive and selective systems for telomerase acting as a potential biomarker [97,98]. Modern micro- and nanofabrication techniques are used to directly produce nanoporous gold array (NPGA) on a substrate. A gold coating was first evaporating on a silicon wafer. A 100 nm thick Au-Ag alloy coating was then sputtered-deposited using an alloy target. After forming a monolayer of polystyrene (PS) beads with a diameter of 460 nm on top of the alloy film, the PS beads were subjected to a timed oxygen plasma treatment to contract, ensuring the separation of neighboring beads. Sputtering and Argon plasma were then used to the sample to transfer the bead pattern into the alloy film. After the pattern transfer, the PS beads were removed using sonication in chloroform. The samples were then immersed in 70% nitric acid to dealloy them. 3D hybrid nanoarchitectures of catalytically assembled tandem G-quadruplexes (G4) were created on the NPGA for monitoring the telomerase activity without any complicated labeling process. This study utilized the highly active surface and good biocompatibility of NPGA which further enhanced the catalytic performance of telomerase. Malachite green (reporter molecule) was then captured by the assembled tandem G4 onto the NPGA for surface-enhanced Raman scattering (SERS) detection. It was seen that compared to the commercial sensing methods, this sensor was 2 orders of magnitude more sensitive and specific toward the active telomerase [99]. Another convenient and controllable way of fabricating nanoporous gold arrays has been shown by the electrodeposition of gold layers on well-ordered nanoporous silicon films. Np-Au hybrid nanocomposites with tunable plasmonic resonance peaks have shown improved SERS sensitivity which can be exploited to create novel sensors and imaging labels [100]. The NPGD are created in this study using a nanosphere lithography-based technique. According to fabrication circumstances, as-prepared NPGDs have an average diameter of 300 nm, a thickness of 75 nm, and a pore size of 8.5 nm. The sub-micron nanoporous gold disk (NPGD) with lithographic patterning has high density hotspots and a wide surface area. NPGDs have plasmonic properties that can be modified by changing their internal nanoporous morphology through controlled dealloying, laser and furnace annealing, and surface changes, in addition to having substantially increased spectroscopic sensing capabilities. Gold nanoparticles of different sizes have been loaded onto NPGD substrates to form a hybrid structure with new plasmonic hot spots due to the coupling between nanoparticles and NPGD [101].

**Figure 5.**
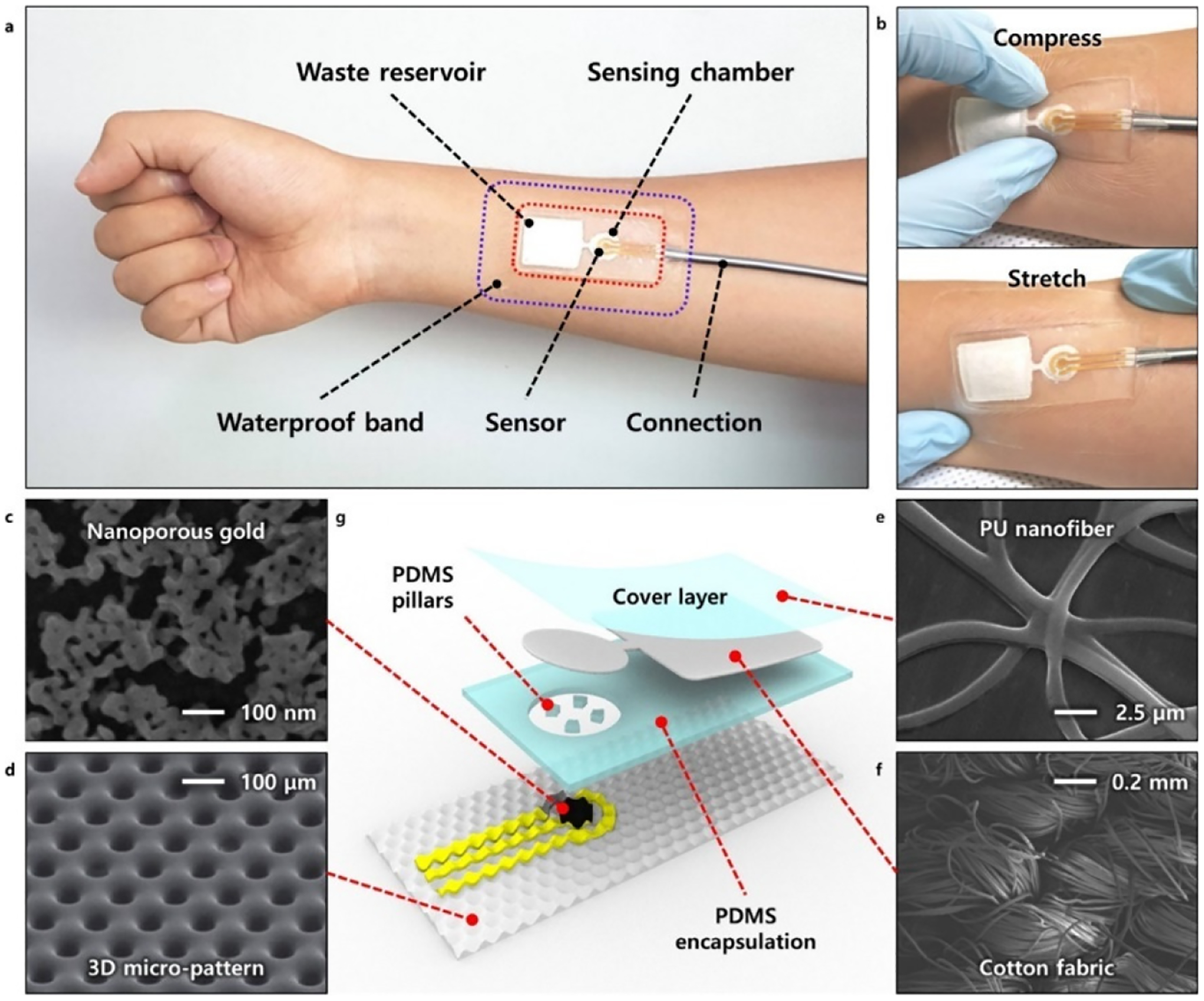
Schematic of stretchable microfluidics-integrated biosensor patch. (**a**) Photograph of fabricated patch mounted on the human body. (**b**) Demonstration of the conformality of the patch to human skin under compression or stretching. FE-SEM images of (**c**) np-Au electrochemical electrode (top view), (**d**) 3D micropatterned PDMS substrate, (**e**) PU nanofiber, and (**f**) stretchable cotton fabric. (**g**) Layered components in the fully stretchable microfluidics-integrated biosensor patch. Reprinted with permission from ref. [103] Copyright 2019, American Chemical Society.

Np-Au-modified biosensors with hybrid structures have been found more promising for meeting the demands of a highly sensitive and reliable biosensor with rapid response and better selectivity. Many related studies have mentioned the synergistic effect of hybrid structures behind this enhanced sensitivity and selectivity. X. Y. Lang et al. (2013) have reported a flexible and self-supported microelectrode having np-Au/cobalt oxide hybrid structure for electrochemical detection of glucose [92]. As per the study, the synergistic approach of gold skeleton, np-Au and cobalt oxide nanoparticles leads to the enhanced oxidation of glucose and thereby resulting in ultrahigh sensitivity. The sensitivity of up to 12.5 mA mM^−1^ cm^−2^ at a very short response time of less than a second was reported by the researchers with a very low detection limit of 5nM. A study performed by Y. Pei et al. (2018) used a hybrid structure of highly surface-roughened np-Au/Au-Sn alloy to increase the performance of a glucose biosensor [102]. They have reported a rapid detection of glucose with a wide linear detection range of 2 μM to 8.11 mM having a low detection limit of 0.36 μM with a signal-to-noise ratio of 3. They have calculated the sensitivity of 4.3746 mA mM^−1^ cm^−2^ and found a good response along with selectivity and reproducibility. C. W. Bae and fellow researchers (2019) have developed wearable continuous glucose monitoring electrochemical biosensors with high performance using a hybrid structure of fully stretchable capillary microfluidics/np-Au [103]. They have fabricated the high-performing electrocatalytic np-Au electrode on a substrate of a stress-absorbing three-dimensional (3D) micro-structured polydimethylsiloxane (PDMS) to develop a highly stretchable, durable, and sensitive sensor to detect glucose in a nonenzymatic manner. They have explained the excellent performance of the integrated biosensor patch for monitoring the sweat glucose level continuously and accurately with high efficiency. Figure 5 shows the design and fabrication of stretchable np-Au which is utilized as a working electrode of the biosensor for the sensitive non-enzymatic glucose-sensing in the presence of interfering species [103].

The selection of least toxic Au nanostructures in biomedical applications is essential for achieving the optimal therapeutic response. Such Au-based nanostructures can be toxic or non-toxic depending on their size, form, surface chemistry, and surface charge [104,105]. Conventional techniques for creating Au nanostructures frequently result in surface contamination from ligands, stabilizers, reducers, or other leftover products that can be hazardous. Additionally, as positively charged particles could undesirably interact with negatively charged DNA, the surface charge of the NPs can be of issue. The size of Au NPs has significantly affected both the biodistribution and excretion pathway. When compared to bigger AuNPs, tiny AuNPs show improved circulation times and a distinct biodistribution. In general, spherical nanoparticles between 15 and 50 nm in size appear to be less harmful, assuming they are suitably coated and devoid of toxic impurities [106,107]. It has been shown that covering Au NPs with albumin makes them more colloidally stable and less likely to interact with plasma proteins. Surface modification of Au NPs with suitable capping agents reduces nanoparticle phagocytosis by the reticuloendothelial system with accumulation in the liver and spleen as a result, lengthening their half-lives in circulation and enabling targeted administration to malignancies [108].

## 4. Conclusions and Prospects

Among the various porous metals, np-Au has stimulated extensive research enthusiasm due to the possibility of tuning the pore size, presence of a long-range bicontinuous ligament structure, chemical stability, biocompatibility, along with easy and quick preparation with high reproducibility. This has made np-Au as one of the most promising materials in analytical chemistry.

This review has selectively summarized the recent research in the therapeutic potential of np-Au used in its bare and surface-modified forms. The research at the interface of biology and np-Au is booming and modern research in nanomedicine is taking advantage of the tunable porous architecture of np-Au by functionalizing its surface with drugs and targeting molecules and modifying pore size, ligament width, inter-ligament distance, and surface charge for selective targeting. Mechanism of action depends on the size of the porous gold carriers along with the capping groups on the surface providing stability and helping in targeted delivery. A separate section has been devoted to discussing the emerging biomedical applications of np-based platforms. Studies related to the fabrication of np-Au-based biosensors using its various forms such as bare np-Au, surface-functionalized np-Au, shape-controlled np-Au, and other np-Au with hybrid structures have been mentioned. Smart nanomaterials range from polymer-based nanoparticles to inorganic metal, metal oxide, and metalloid nanoparticles. Here, we describe the surface manipulation with polymers and enzymes for the application of np-Au in biosensor field. Hybrid structures involving np-Au along with graphitic carbon nitride, metal oxides, alloy particles, biomolecules like DNA, peptides, and other small dye molecules have been found more promising for meeting the demands of a highly sensitive and reliable biosensor with rapid response and better selectivity.

We foresee great potential in np-Au to be used as a functional medical coating and in point-of-care medical devices. Nanotherapeutics are steadily proving to be superior to traditional methods in terms of their enhanced therapeutic efficacy, diagnostic sensitivity, simplified dosing schedule, and improved patient compliance [109]. Nanoporous gold-based structures are propelling the rapid development in the emerging fields of targeted and controlled drug delivery platforms, biomedical imaging systems, portable electronics useful for biosensing of pathogens, (photo)hyperthermia, and gene therapy [110]. Recent years have witnessed considerable research on implants based on np-Au due their fascinating pore size-dependent properties, and structural periodicity. The possibility of surface modification and optimizing pore size and ligament width can open doors in the future for creating implants of controlled release profiles [17]. The dendrimer-based nanomedicines are geometrically growing. The amplification property due to their branched structure and multivalency effect provide the chance to detect diseases in their early stages. In the next decade, we feel that the combined structures formed from porous gold and biocompatible dendrimers will lead to breakthroughs in therapeutic and diagnostic applications of np-Au [109]. An evolving new field of theranostics (integration of imaging and therapy) is making use of emerging materials and nanotechnologies to rationally design platforms for the simultaneous imaging and therapy of cancer. There is a great potential of using np-Au-hybrid materials to tap on the synergistic effect between the plasmon resonance of np-Au and the other components for getting an enhanced surface plasmon resonance effect and loading capability of contrast agent [111].

Due to high surface area, superior conductivity, and biocompatibility, nanoporous gold is a desirable material for biosensing, catalysis, fuel cells, and drug delivery. However, the universal application np-Au in research in industry is currently constrained by a number of factors [112]. The largest barrier to the widespread use of NPG, in the first place, is the expense of manufacture. This is because, as a precious metal, NPG has a higher use cost than other nanoporous materials like zeolite, mesoporous TiO_2_, porous silica, etc. In this regard, developing more efficient methods to achieve the best np-Au performance with the least amount of material usage is a significant research direction. It is projected that research in this area will increase and continue, with an emphasis on manufacturing optimization to improve the targeting of molecules of interest even further [113,114]. The mechanical strength of np-Au needs to be enhanced, which is the second issue. Both the remarkable characteristics of np-Au and its fragility are due to its porous nanoscale structure. Under mechanical stress, particularly NPG thin films, are easily fractured. The diffusion restriction within the pores is the third issue. In practical applications, finding a balance between specific surface area and diffusion efficiency is a challenge. Finally, it is necessary to take into account np-Au effects on the environment. For instance, np-Au created by de-alloying Au-Ag alloy will be hazardous to the environment. Additionally, issues like the bulk manufacture of np-Au must be addressed [53].

More work should be put into developing and implementing np-Au based devices for practical sensing applications in environmental monitoring, biomarker detection, and food safety & quality control since there is still a substantial gap between these studies and their implementation in industry [115]. In the realm of porous metal and porous materials, we can anticipate several years of technological growth and ground-breaking scientific findings due to the ongoing improvements in manufacturing and characterization [116].

Despite having drawbacks like high manufacturing costs, the inability to mass produce, low mechanical strength, and inadequate environmental resistance, np-Au still has a significant amount of room for development and a wide range of opportunities in the production of miniaturized, integrated, multi-functional, intelligent, and portable devices due to the rising demands in a variety of industries, including the food, medical, environmental, and basic scientific research. One of them, in the current environment where infectious diseases regularly arise, deserves our attention and investigation. It is the superior pathogen detection capabilities of np-Au based biosensors at the nucleic acid, protein, and even whole-cell levels. A confluence of various sectors, technologies, and disciplines will continue to support the implementation of np-Au on the basis of further theoretical investigation [53].
